# Efficacy of autologous platelet-rich plasma use for arthroscopic meniscal repair: A randomized trial protocol

**DOI:** 10.1097/MD.0000000000023422

**Published:** 2020-11-25

**Authors:** Hongchang Yu, Rongrong Tan, Baozhen Lou, Dingshan Xue

**Affiliations:** a Department of Orthopaedics; b Department of Anesthesiology, PLA Army 80th Group Military Hospital, Shandong, China.

**Keywords:** arthroscopic meniscal repair, pain control, platelet-rich plasma, randomized controlled trial, study protocol

## Abstract

**Background:**

Meniscus tear is one of the most familiar orthopedic injury, and it is also the leading cause of the dysfunction of knee joint. Recent efforts to improve the success rate of the meniscus repair surgery involve the addition of platelet-rich plasma (PRP). The aim of our experiment is to assess the clinical effects of arthroscopic repair of meniscal tears without or with PRP.

**Methods:**

This is a randomized and parallel-group superiority study. The study protocol is approved through the review committee of the corresponding institutions in PLA Army 80th Group Military Hospital. All patients will provide written informed consent to participate in the study. We implement our investigation on the basis of the ethical standards outlined in the Helsinki Declaration of 1964 and then report our outcomes according to the CONSORT statement of 2010. All the patients follow a same rehabilitation program. Patients are assessed at baseline (day before operation), 12 months and 24 months after the last time of injection; outcome assessments involve Ikeuchi score, Lysholm score, and the visual analogue scales for failure and pain rate. *P* value less than .05 indicates that there is statistical significance.

**Results:**

We suppose that arthroscopic PRP repair of meniscus tears results in improved pain and functional results owing to the release of bioactive molecules that may affect the healing of meniscus.

**Trial registration:**

This study protocol was registered in Research Registry (researchregistry6175).

## 1 Introduction

Meniscus tear is one of the most familiar orthopedic injury, and it is also the leading cause of the dysfunction of knee joint, due to the role of meniscus provides shock absorption and joint stability, and helps to prevent the degeneration of articular cartilage.^[1–5]^ It is estimated that approximately 4 million meniscus arthroscopies are carried out worldwide each year.^[6]^ The long-term impacts of the meniscus injury are severe, generally resulting in early symptomatic osteoarthritis and dysfunction. The injuries of meniscus are heterogeneous in etiology and morphology. Some tears do not heal and require the meniscus resection.^[7–10]^ Recently, there have been changes that restrict meniscus resection and perform meniscus repair when needed. In recent years, the incidence of meniscus repair has increased by more than 11%.^[11–13]^

Recent efforts to improve the success rate of the meniscus repair surgery involve the addition of platelet-rich plasma (PRP). It has been promoted as a kind of desire product of autologous biological blood derivative, which can be applied externally to a variety of tissues, where it can release the platelet-derived growth factors with high concentrations to promote wound healing, tendon healing, and bone healing.^[14–17]^ The standard for the repair of meniscus includes a vertical mattress suturing configuration from the inside out. PRP clots can be sutured to the site of repair and transported under the condition of direct visualization.^[18]^ Fundamental scientific researches have indicated that PRP for the tendon repair can increase the failure force, enlarge the tendon callus and enhance the proliferation of tendon cell. To our knowledge, limited researches have explored the clinical efficacy of arthroscopic PRP-enhanced repair of the meniscus tears.^[19–21]^

Hence, the aim of our experiment is to assess the clinical effects of arthroscopic repair of meniscal tears without or with PRP. We suppose that arthroscopic PRP repair of meniscus tears results in improved pain and functional results owing to the release of bioactive molecules that may affect the healing of meniscus.

## 2 Materials and methods

### 2.1 Study design

This is a randomized and parallel-group superiority study. The study protocol is approved through the institutional review committee in PLA Army 80th Group Military Hospital, and it will be published before the first patient registration. We implement our investigation on the basis of the ethical standards outlined in the Helsinki Declaration of 1964 and then report our outcomes according to the CONSORT statement of 2010. All patients will provide written informed consent to participate in the study. The protocol has been registered with the Research Registry under the number researchregistry6175 and is publicly accessible.

### 2.2 Patients

In this study, patients who meet the following criteria will be included: patients aged between 18 and 55 years; patients with peripheral unstable tears; MRI full vertical longitudinal tear length greater than 10 mm; patients with meniscus injury 1 month to 18 months before the surgery; Cooper zone 2 meniscus lesions (more than 4 mm from the edge). Patients with more than 18 months of preoperative meniscus injury, changes in arthritis (Kellgren-Lawrence score greater than 2); meniscus lesions in Cooper zone 0–1, not exceeding 4 mm from the edge; existence of crystals or degeneration in meniscus; inflammatory diseases and accompanying surgical operations (e.g., ligament reconstruction, microfracture, trephing, and fracture fixation) were excluded.

### 2.3 Randomization

The research assistant, who does not participate in any subsequent studies or communicate with other members of the research team throughout the study, prepare the same number of envelopes for each experimental group through utilizing the computerized random number generator. He prepares 100 same sequentially opaque, numbered, bound, and sealed envelopes; 50 of them included the instructions for the PRP group and 50 are for the placebo group. All the envelopes are kept in the archives of the principal researcher.

### 2.4 Techniques and devices

All the arthroscopic procedures are implemented via utilizing intraregional techniques. All the operations are conducted with a senior surgeons and all patients undergo the identical surgical procedures. The arthroscopic system involves a system of Dyonics 25 fluid management acts as the circulation system, Fast-Fix 360 for all the internal inside sutures, the Clear-Trac flexible 4.5 mm sleeve to output the circulating fluid, a device to inject the PRP to the joints as well as the Meniscus for semi-external sutures Mender II.

### 2.5 PRP preparation

The PRP is acquired via utilizing the Arthrex ACP Dual Syringe System. And such system consists of a 10 ml external syringe, which connects to the commercial 5 ml syringe. The autologous blood (10 ml) was drawn from the anterior cubital vein with an external syringe and then it is put into an Arthrex Centrifuge. Subsequently, the blood autologous is centrifuged at a speed of 1500 rpm for 5 minutes. During the in vitro blood treatment, acid-citrate-dextrose solution (2 ml) is utilized to prevent coagulation. Under the sterile conditions, the system allows the transfer of supernatant from an external syringe (10 ml) to a syringe (5 ml). The average yield is in the range of 2 to 5 ml.

### 2.6 Postoperative rehabilitation

All the patients follow a same rehabilitation program. After the meniscus repair under arthroscopy, knee joint extension brace is required, with limited flexion within 10 weeks. The complete weight-bearing is allowed immediately after surgery for the total knee extension, and physical therapy is given to the patients. A locking knee brace is applied every thirty degrees for 2 weeks. And light operation is permitted after 3 months.

### 2.7 Outcome evaluations

Patients are assessed at baseline (day before operation), 12 months and 24 months after the last time of injection; outcome assessments involve Ikeuchi score, Lysholm score, and the visual analogue scales for failure and pain rate. Failure includes the symptoms of swelling or locking or requiring the repeated arthroscopy and/ or the pain of patients joints. The result data is recorded via the plastic surgeons by telephone or in person, and the orthopedic surgeons is unaware about the treatment underwent by patients.

### 2.8 Statistical analysis

In accordance with the prior power analysis, the patients number followed up for 3 years was sufficient to verify the hypothesis of preliminary study. The minimal clinically significant difference in the risk of the failure of meniscal repair has not been published; in our opinion, a 50% reduction in the risk of the failure of meniscal repair owing to PRP would be sufficient to justify the routine clinical application of PRP. Assuming that there is an estimated 20% risk of the failure of meniscal repair within 3 years after surgery without the PRP, we determined that minimum size of sample (alpha = 0.05 and 80% power) required to test a 50% reduction in the risk of the failure of meniscal repair associated with PRP is n = 50 with PRP and n = 50 without PRP. The statistical analysis was implemented via the independent experts and was not took part in the research program. The range of mean values and median values were presented. The non-paired *t* test was applied for the numerical data of normally distribution. Where appropriate, the non-parametric simulation was utilized. The comparison of categorical variables was carried out by x^2^ test. *P* value less than .05 indicates that there is statistical significance.

## 3 Discussion

In the existing literature, the clinical applications of PRP in the repair of meniscus is more varied, with distinct indications, preparation techniques of PRP and results. The aim of our experiment is to assess the clinical effects of arthroscopic repair of meniscal tears without or with PRP. We suppose that arthroscopic PRP repair of meniscus tears results in improved pain and functional results owing to the release of bioactive molecules that may affect the healing of meniscus. The study may possess several limitations, involving the lack of long-term follow-up and the small patients number. However, this study will guide and clarify our assignments, and the final outcomes and conclusion will further enrich the clinical knowledge in the literature.

## Author contributions

**Conceptualization:** Baozhen Lou.

**Data curation:** Hongchang Yu.

**Formal analysis:** Hongchang Yu, Rongrong Tan, Baozhen Lou.

**Funding acquisition:** Dingshan Xue.

**Investigation:** Hongchang Yu, Rongrong Tan, Baozhen Lou.

**Methodology:** Dingshan Xue.

**Resources:** Dingshan Xue.

**Software:** Rongrong Tan.

**Supervision:** Dingshan Xue.

**Validation:** Dingshan Xue.

**Visualization:** Dingshan Xue.

**Writing – original draft:** Hongchang Yu

**Writing – review & editing:** Dingshan Xue.
